# Induction of spatial anxiety in a virtual navigation environment

**DOI:** 10.3758/s13428-022-01979-1

**Published:** 2022-10-12

**Authors:** Alice Oliver, Tim Wildschut, Matthew O. Parker, Antony P. Wood, Edward S. Redhead

**Affiliations:** 1 School of Psychology, University of Southampton, Highfield, Southampton, England, SO17 1BJ UK; 2 School of Pharmacy and Biomedical Science, University of Portsmouth, Portsmouth, UK

**Keywords:** Spatial anxiety, Navigation, Spatial disorientation, Virtual environments

## Abstract

Spatial anxiety (i.e., feelings of apprehension and fear about navigating everyday environments) can adversely impact people’s ability to reach desired locations and explore unfamiliar places. Prior research has either assessed spatial anxiety as an individual-difference variable or measured it as an outcome, but there are currently no experimental inductions to investigate its causal effects. To address this lacuna, we developed a novel protocol for inducing spatial anxiety within a virtual environment. Participants first learnt a route using directional arrows. Next, we removed the directional arrows and randomly assigned participants to navigate either the same route (*n* = 22; control condition) or a variation of this route in which we surreptitiously introduced unfamiliar paths and landmarks (*n* = 22; spatial-anxiety condition). The manipulation successfully induced transient (i.e., state-level) spatial anxiety and task stress but did not significantly reduce task enjoyment. Our findings lay the foundation for an experimental paradigm that will facilitate future work on the causal effects of spatial anxiety in navigational contexts. The experimental task is freely available via the Open Science Framework (https://osf.io/uq4v7/).

Spatial disorientation has negative practical and emotional consequences (Lynch, 1960) and can undermine confidence in performing wayfinding tasks, resulting in spatial anxiety or feelings of apprehension and fear about environmental navigation (Lawton, 1994). Spatial anxiety is a domain-specific construct or “surface” trait, referring to negative emotions that arise exclusively within spatial contexts (Lyons et al., 2018; Malanchini et al., 2017; McKheen, 2011; Vieites et al., 2020). Broader constructs such as general anxiety are domain-general or “personality” traits. General anxiety is associated with neuroticism, which denotes negative affectivity and vulnerability to stress (Cox et al., 1999). Various anxiety constructs (e.g., general, mathematics, test, spatial), although interrelated, are distinct (Alvarez-Vargas et al., 2020; Malanchini et al., 2017; McKheen, 2011), with each construct comprising unique genetic factors (Malanchini et al., 2017). Individual differences in spatial anxiety are not fully explained by general anxiety (Alvarez-Vargas et al., 2020). For example, general anxiety, as opposed to spatial anxiety, does not relate to navigation ability (Walkowiak et al., 2015), indicating that people high in general anxiety are not necessarily high in spatial anxiety, and vice versa. Thus, domain-general and domain-specific anxiety should be treated separately.

It has been widely accepted that anxiety hinders cognitive performance (Maloney et al., 2014; Moran, 2016; Sandi, 2013). Compared to other types of anxiety (i.e., general, mathematics, test), spatial anxiety is arguably the most understudied. Past research has found that self-reported spatial anxiety is negatively correlated with navigation performance, in terms of reducing speed and increasing errors (for a review see, Coluccia & Louse, 2004; Hund & Minarik, 2006; Walkowiak et al., 2015). Prior correlational studies have operationalized spatial anxiety using self-report questionnaires (Lawton, 1994, 1996). However, inferences drawn from correlational designs are limited, and the direction of causality between relatively poor navigation and relatively high spatial anxiety remains unclear (Weisberg & Newcombe, 2018). Experimental procedures to directly induce spatial anxiety within a spatial task are currently lacking. To address this lacuna, we developed a novel protocol for manipulating spatial anxiety within a virtual environment.

Virtual environments offer flexible, interactive design features which can be controlled and displayed from a 3D first-person perspective (Richardson et al., 1999). Virtual platforms are also safe; if the user makes navigational errors, harm is minimal. Although virtual environments provide visual stimuli, they do lack vestibular, proprioceptive, and efferent information which is present during real-world navigation. Despite this, the acquisition of spatial knowledge can be simulated to resemble real-world settings (Ruddle et al., 1997). Several studies have shown that performance in the real world is comparable to performance in virtual tasks, including in clinical populations such as people with mild cognitive impairment, Alzheimer’s disease, and schizophrenia (Aubin et al., 2018; Coutrot et al., 2019; Cushman et al., 2008; Kalová et al., 2005), making virtual environments a cost-effective and ecologically valid tool. Moreover, researchers have used virtual environments to induce transient affective states, such as sadness, relaxation, joy, and fear (Baños et al., 2012; Felnhofer et al., 2015; Riva et al., 2007; Toet et al., 2009). Three studies in particular altered environmental conditions to induce fear. Riva et al. (2007) and Felnhofer et al. (2015) modified the audio cues and intensity of lighting in a virtual park and successfully evoked a fearful state. However, Toet et al. (2009) found that active exploration of a darkened (compared to a brightened) virtual village did not elicit fear, even after an acute stress task (i.e., Trier Social Stress Test).

Lynch (1960) identified getting lost as an anxiety-provoking experience that is intimately connected to one’s sense of emotional security. Although researchers have exposed participants to adverse sensory stimuli in virtual environments, to date no study has simulated the experience of becoming lost. Here, we aim to fill this gap and, by so doing, develop an experimental paradigm for investigating spatial anxiety. We implemented our spatial-anxiety manipulation in a virtual route-learning task and assessed its impact on transient (i.e., state-level) spatial anxiety and affect.

## Method

### Participants and design

Forty-six University of Southampton undergraduate students (31 women, 13 men) took part in a 40-min experiment in return for course credit. The experimenter terminated the study early for two participants (one participant felt unwell and one experienced a computer error). We excluded these participants from all analyses. Participants’ age ranged from 18 to 29 years (*M* = 20.20, *SD* = 2.24). Participants’ ethnicities were: White British (*n =* 34), other white background (*n =* 4), Caribbean (*n =* 2), Bangladeshi (*n =* 1), Indian (*n =* 1), Pakistani (*n* = 1), African (*n* = 1), other Asian background (*n* = 1), and other mixed background (*n* = 1). All participants had normal or corrected to normal (soft contact lenses or glasses) vision. We randomly assigned participants to one of two conditions: spatial anxiety (*n* = 22) and control (*n* = 22). We conducted a power analysis using G*Power 3.1 (Faul et al., 2007). Our key objective was to demonstrate the effectiveness of the spatial-anxiety manipulation. As such, the primary outcome variable was transient or state-level spatial anxiety and, based on pilot testing, we anticipated a large effect (*d* = 1.00). The power analysis indicated a requisite sample size of 34 to achieve power equal to .80 (two-tailed alpha = .05). We exceeded this target to hedge against attrition. This study received ethical approval from the University of Southampton Ethics Committees (2019-31054).

### Virtual environment

**Fig. 1:**
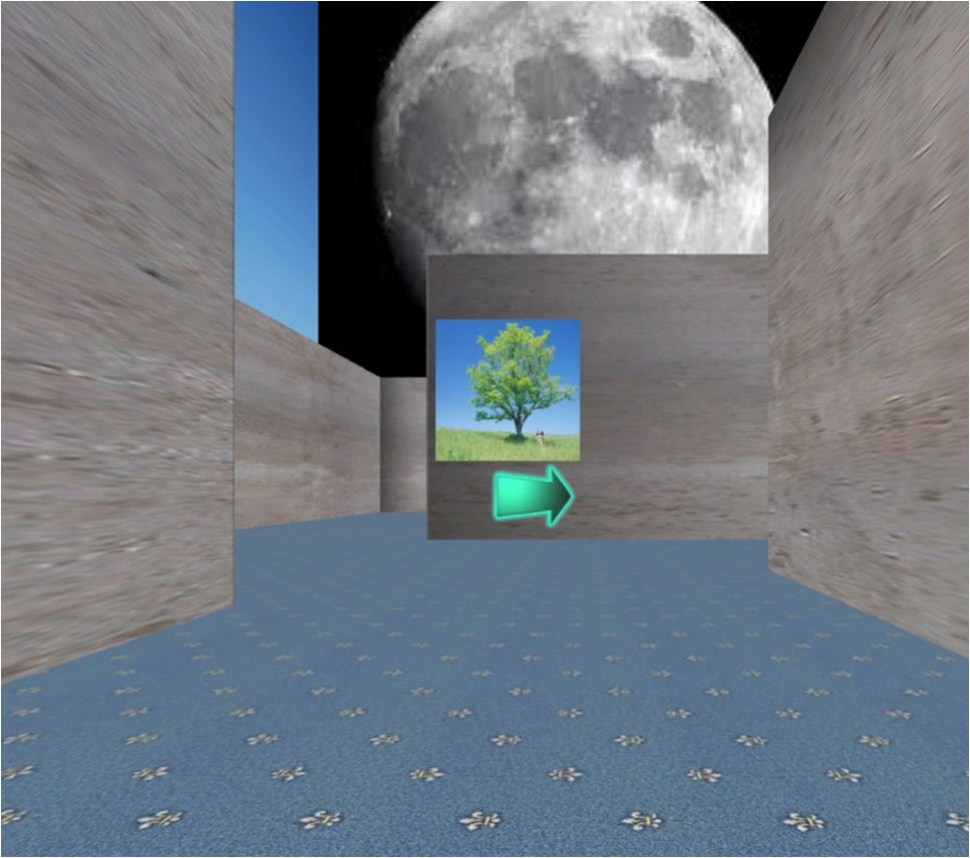
Virtual maze presentation from a participant’s perspective

We used the Unity development platform to create a Windows desktop application of a virtual 3D maze environment, which is freely available for download. Exploration through the environment presented a first-person perspective (Fig. 1). The program restricted the participant’s view to a static plane, in that they could not maneuver their gaze up or down. To control movement through the maze, participants used the arrows keys “FORWARD,” “BACKWARD,” “LEFT,” and “RIGHT” on the keyboard. The maze route only included perpendicular turns. Along the maze route, the walls displayed ten local landmarks. The local landmarks comprised 2D colorful pictorial images, such as apple, tree, bus, or wallpaper patterns. Outside the maze, but within distal view, four global landmarks were located at each cardinal reference point. The global landmarks depicted a hot air balloon, moon, high-rise buildings, and a mountain-range scene. The local and global landmarks functioned as spatial reference points to aid navigation.

### Procedure and materials

**Fig. 2:**
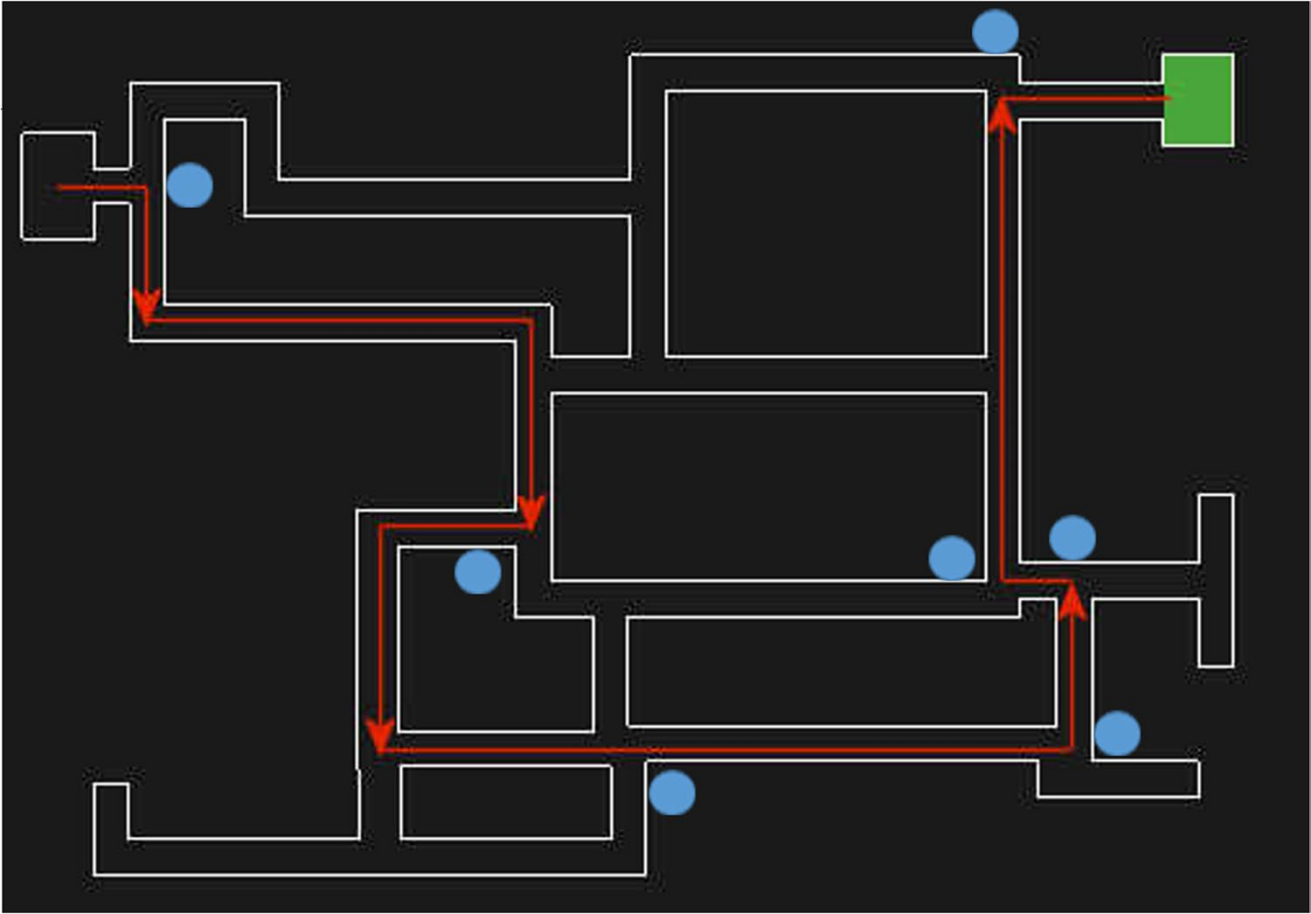
Schematic of the Maze 1 layout and route. The *red arrows* illustrate the specified route and the *green box* indicates the end destination. The *blue circles* indicate the position of pictures

After reading the information sheet, participants provided consent and then completed demographic information. Next, they started the route-learning task. This task consisted of three training trials and one test trial. On the training trials, directional arrows were present in the maze, and participants followed these arrows to navigate from a starting point to an end destination. The training route involved ten turns (Maze 1; Fig. 2).

**Fig. 3:**
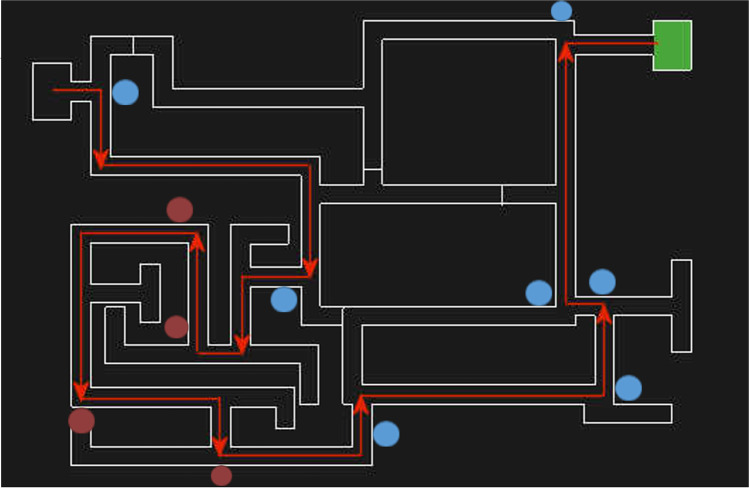
Schematic of the Maze 2 layout and route for the test trial in the route-learning task. The *red arrows* illustrate the specified route and the *green box* indicates the end destination. The *blue circles* indicate the position of pictures and the *red circles* indicate the new pictures introduced as part of the spatial-anxiety manipulation

Next, participants completed the test trial, where the directional arrows were absent. Participants in the control condition navigated the same maze (Maze 1) as in the training trials. Participants in the spatial-anxiety condition navigated Maze 2 (Fig. 3), a variation of the maze used in training. In Maze 2, the route from the starting point to the fifth turn was the same as in Maze 1. When participants passed the fifth turn, however, the maze presented additional paths with dead ends and four unfamiliar local landmarks. After the ninth turn, the route returned to the original layout and appearance of Maze 1. If participants were unable to complete the critical trial within 4 min, the experimenter guided them to the end destination. Immediately after the route-learning task, participants completed the following measures.

#### State spatial anxiety

We adapted the Spatial Anxiety Scale (Lawton & Kallai, 2002) to measure transient, state-level spatial anxiety. Participants rated (1 = *not at all anxious*, 5 = *very anxious*) how anxious they would feel “Right now, that is, at this present moment” in relation to eight hypothetical navigation scenarios (e.g., “Finding my way to an appointment in an unfamiliar area of a city or town,” “Trying a new route that I think will be a shortcut, without a map”; α = .91, *M* = 3.02, *SD* = 0.91).

#### Task experience

Participants evaluated (1 = *strongly disagree*, 6 = *strongly agree*) their task experience on 15 items (e.g., “The navigation task was enjoyable”), which we submitted to an exploratory factor analysis. We used parallel analysis (Horn, 1965) and minimum average partial correlation (MAP) analysis (Velicer, 1976) to determine the number of factors to retain. In addition, we examined eigenvalues and the proportion of common variance explained by each factor. All criteria pointed to a two-factor solution (see Table 1), with the two main factors jointly accounting for 80% of the common variance. The obliquely rotated (promax) factor pattern displayed simple structure. The items “stressed,” “difficult,” “frustrated,” “doubt my ability,” “anxious,” “something I am good at” (negatively), and “easy” (negatively) loaded exclusively on the first factor (factor loading > .50), which we labeled Task Stress (α = .90, *M* = 3.40, *SD* = 1.08). The items “fun,” “engaging,” “interesting,” “exciting,” “enjoyable,” “something I would like to do again,” “boring” (negatively), and “tedious” (negatively) loaded exclusively on the second factor, which we labeled Task Enjoyment (α = .88, *M* = 4.62, *SD* = 0.72). A debriefing concluded the experiment.

**Table 1:** Factor analysis for the task experience questionnaire (*N* = 44): rotated factor pattern

Items	Factor loading	
	1	2
Factor 1: Task stress		
7. Stressed	.85	-
11. Difficult	.81	-
9. Frustrated	.76	-
10. Doubt my ability	.66	-
8. Anxious	.65	-
15. Good at	– .80	-
4. Easy	– .80	-
Factor 2: Task enjoyment		
2. Fun	-	.85
13. Engaging	-	.83
14. Interesting	-	.81
3. Exciting	-	.78
1. Enjoyable	-	.68
12. Do again	**-**	.59
5. Boring	-	– .55
6. Tedious	-	– .72

*Note*. Factor loadings smaller than .50 are omitted

#### Control variable

We administered two trait-level measures assessing dispositional individual differences in spatial anxiety. Both scales assessed how anxious participants feel in general (rather than at the present moment) in relation to hypothetical navigation scenarios. We did not expect that our momentary induction of spatial anxiety would alter dispositional spatial anxiety. Rather, we controlled for this trait to ascertain that any effects of the spatial-anxiety induction were not due to (or obscured by) pre-existing differences between conditions in dispositional spatial anxiety. Random assignment guards against such pre-existing differences but does not rule them out. The revised Spatial Anxiety Scale (Lawton & Kallai, 2002) comprises eight items (e.g., “Finding my way to an appointment in an unfamiliar area of a city or town”) that were rated on a five-point scale (1 = *not at all anxious*, 5 = *very anxious*; α = .81, *M* = 2.86, *SD* = 0.67). The Spatial Anxiety Questionnaire (Malanchini et al., 2017) includes ten items (e.g., “Finding your way around an intricate arrangement of streets”) that were rated on a five-point scale (1 = *not at all anxious*, 5 = *very anxious*; α = .80, *M* = 2.40, *SD* = 0.59). We pooled the 18 items across both scales to create an overall index of dispositional spatial anxiety (α = .89, *M* = 2.67, *SD* = 0.67).

## Results

### State spatial anxiety

Participants in the spatial-anxiety condition (*M* = 3.47, *SD* = 0.77) reported significantly higher levels of state-level spatial anxiety than participants in the control condition (*M* = 2.57, *SD* = 0.82), *t*(42) = 3.75, *p* < .001, *d* = 1.13. Further, the mean spatial-anxiety score in the spatial-anxiety condition (*M =* 3.47, *SD =* 0.77) significantly exceeded the scale midpoint (= 3), *t*(21) = 2.86, *p* = .009. The induction of spatial anxiety was successful, both in comparison to the control condition and relative to the scale midpoint.

### Task experience

Participants in the spatial-anxiety condition (*M* = 4.24, *SD* = 0.74) scored significantly higher on the Task Stress scale than participants in the control condition (*M* = 2.56, *SD* = 0.62), *t*(42) = 8.18, *p* < .001, *d* = 2.47. The spatial-anxiety (*M* = 4.64, *SD* = 0.64) and control (*M* = 4.60, *SD* = 0.81) conditions did not differ significantly on Task Enjoyment, *t*(42) = 0.18, *p* = .860, *d* = 0.05.

We also tested the effect of the spatial anxiety manipulation on each task-experience item, using a Bonferroni-adjusted alpha level of .0033 (.05/15). We present the results in Table 2, in descending order of effect size. Participants in the spatial-anxiety condition (compared to controls) were more frustrated, stressed, and anxious. They were also more likely to indicate that the route-learning task was difficult and made them doubt their ability, and less likely to think the task was easy and something they were good at.

**Table 2:** Means and standard deviations for affect items by condition

	Spatial anxiety	Control	*t*	*p*	*d*
	*M* (*SD*)	*M* (*SD*)			
Frustrated	3.95 (1.22)	1.64 (0.79)	7.51	< .001	2.26
Difficult	4.27 (1.16)	2.50 (0.80)	5.89	< .001	1.78
Easy	2.82 (1.10)	4.36 (0.79)	– 5.36	< .001	– 1.62
Stressed	4.14 (0.94)	2.55 (1.10)	5.15	< .001	1.55
Something I am good at	2.68 (1.36)	4.32 (0.78)	– 4.90	< .001	– 1.48
Doubt my ability	4.73 (1.20)	2.95 (1.36)	4.58	< .001	1.39
Anxious	4.09 (1.11)	3.00 (1.20)	3.14	.003	0.94
Engaging	5.05 (0.79)	4.55 (0.96)	1.89	.066	0.57
Interesting	5.00 (0.87)	4.59 (0.96)	1.48	.146	0.45
Enjoyable	4.41 (0.73)	4.73 (0.83)	– 1.35	.184	– 0.41
Something I’d do again	4.00 (1.07)	4.23 (1.11)	– 0.69	.493	– 0.21
Exciting	4.09 (1.23)	3.91 (1.41)	0.46	.651	0.13
Fun	4.32 (0.89)	4.45 (1.10)	– 0.45	.654	– 0.13
Boring	2.05 (1.05)	2.09 (0.81)	– 0.16	.873	– 0.05
Tedious	2.22 (1.02)	2.22 (1.06)	0.00	1.00	0.00

### Controlling for dispositional spatial anxiety

As intended (by random assignment), participants in the spatial-anxiety (*M* = 2.71, *SD* = 0.69) and control (*M* = 2.64, *SD* = 0.67) condition did not differ on dispositional spatial anxiety, *t*(42) = 0.34, *p* = .733, *d* = 0.10. When we repeated our analyses with the addition of dispositional spatial anxiety as a covariate, effects of the spatial-anxiety manipulation were essentially unchanged. Results did, however, reveal an important additional finding—dispositional spatial anxiety was positively and significantly associated with transient, state-level spatial anxiety in the navigation task, b* = .49, *t*(41) = 4.45, *p* < .001. This provides construct validation for the state-level spatial anxiety measure (Campbell & Fiske, 1959).

## Discussion

Results supported the effectiveness of our spatial-anxiety induction. Participants in the spatial-anxiety condition, who navigated a maze in which we had surreptitiously introduced unfamiliar elements, reported higher levels of transient spatial anxiety, both in comparison to control participants and relative to the scale midpoint. Task evaluations revealed that the spatial-anxiety induction evoked a mix of stress, anxiety, frustration, and doubt in one’s spatial ability. These results are in line with previous studies that successfully used virtual environments to trigger emotions, such as sadness, relaxation, joy, and fear (Baños et al., 2012; Felnhofer et al., 2015; Riva et al., 2007). Prior virtual designs altered visual and audio features (e.g., darkness, unpleasant noises). Our task is novel in that we simulated the experience of becoming lost—an adverse spatial-related experience (Lynch, 1960).

We demonstrated that virtual platforms are an effective tool for inducing emotions related to navigation. Virtual environments present a life-like interface and the acquisition of spatial knowledge in virtual environments closely resembles real-world navigation (Coutrot et al., 2019; Cushman et al., 2008; Hegarty et al., 2006; Richardson et al., 1999; Ruddle et al., 1997), thus strengthening ecological validity. Further, virtual-environment technology can be readily implemented on personal computers without requiring specialist equipment and can be shared with other researchers and laboratories (Wiener et al., 2020). The protocol we have described here can be accessed free of charge and without restriction: https://osf.io/uq4v7/.

Past research has assessed the effects of acute, generalized anxiety or fear on spatial navigation by using context-irrelevant stressors such as the Trier Social Stress Test (Toet et al., 2009), threat of shock technique (Cornwell et al., 2012), the cold pressor test (Duncko et al., 2007), and a restricted breathing exercise (Ruginski et al., 2018). To date, however, no studies have implemented specific experimental inductions of spatial anxiety. A direct manipulation of spatial anxiety will allow future researchers to examine its effect on spatial cognition and related constructs, such as motivation to explore, navigation experience, strategy preferences, and spatial confidence. Incorporating spatial-anxiety inductions in future studies will help to disambiguate the causal direction of the relation between spatial anxiety and navigation ability, as well as identify mediating mechanisms. Clarifying such mechanisms could inform training programs designed to improve spatial skills (Lovden et al., 2011; Uttal et al., 2013), especially for spatially anxious navigators.

### Limitations and future directions

We acknowledge several limitations. First, our sample lacked representative diversity in gender, race, and age. Future studies should validate the spatial-anxiety induction in more diverse samples to assess its generalizability. Second, although our manipulation successfully induced spatial anxiety, we cannot rule out that it also heightened general anxiety. Domain-specific and general anxiety constructs are only modestly correlated (Alvarez-Vargas et al., 2020, McKheen, 2011) and this limited overlap is primarily due to genetic rather than environmental factors (Malanchini et al., 2017). Nonetheless, future research should assess the specificity of our spatial-anxiety induction to ascertain that its effects are uniquely attributable to spatial, and not general, anxiety. An important next step, then, is to include measures of general anxiety (Löwe et al., 2008; Ree et al., 2008), as well as physiological parameters (e.g., increased heart rate, reduced heart rate variability [Howell & Hamilton, 2022], elevated skin conductance levels [Murty et al., 2011]). By so doing, future studies could strengthen the current findings and enhance our understanding of the specific effects of spatial anxiety. Third, our spatial anxiety procedure is a composite manipulation, in that it introduces (1) a more complex maze route and (2) breaks down established contingencies (i.e., pairings between learnt cues and associated turns at junctions). Future studies should address whether spatial anxiety within the maze results from altering the maze complexity, disrupting cue pairings, or both. Doing so would disambiguate the spatial modification accountable for triggering spatial anxiety.

The new spatial-anxiety manipulation has potential future applications. Strong spatial skills are key for success in science, technology, engineering, and mathematics (STEM) fields (Kell et al., 2013; Wai et al., 2009). Uttal et al. (2013) outlined the malleable yet transferable nature of spatial skills, which is encouraging for researchers keen to help those who are spatially anxious. So far, interventions alleviating domain-general anxiety have proven unsuccessful when applied to specific contexts (e.g., mathematics anxiety; Sharp et al., 2000; Zettle, 2003). Better understanding of spatial anxiety may help develop targeted, domain-specific interventions (Malanchini et al., 2017), which could enhance spatial skills and, in turn, diversify participation in STEM fields. Additionally, investigating the causal effect of spatial anxiety on navigation ability promises to improve our understanding of populations that experience such difficulties in day-to-day life. For example, people living with dementia of the Alzheimer’s type frequently experience spatial anxiety (Chiu et al., 2004; Davis & Veltkamp, 2020; Mahoney et al., 2000; Tu & Pai, 2006). Enhanced insight into the causal effects of spatial anxiety could inform interventions to combat the psychological and practical consequences associated with impaired navigation in this and other vulnerable populations.
